# AI for Alzheimer's Caregiving: Supporting Spouses Through Changing Marital Realities

**DOI:** 10.1002/alz70858_103399

**Published:** 2025-12-25

**Authors:** Erting Sa

**Affiliations:** ^1^ University at Albany, SUNY, Albany, NY, USA

## Abstract

As Alzheimer's Disease progresses, individuals gradually lose access to their autobiographical memories, shared histories, and the ability to recognize their loved ones, profoundly affecting the marital intimacy and interdependence that define spousal relationships. This abstract proposes the potential of artificial intelligence (AI) as a memory‐preserving and meaning‐making companion for spouse caregivers navigating the ambiguous loss associated with their loved one's cognitive decline.

By integrating natural language processing, machine learning, and digital archiving, an AI‐driven system could store and organize key life events, personal narratives, shared symbols and memories, and intimate conversations that have shaped the couple's relationship. The AI would function as an interactive repository, enabling caregivers to access and revisit their spouse's recorded thoughts, voices, and reflections, thereby preserving the emotional essence of their bond even as cognitive decline progresses.

This technology could offer personalized support through reminiscence‐based interactions, guiding caregivers through past conversations and adapting responses based on prior exchanges. Additionally, it could generate therapeutic storytelling experiences, reinforcing the caregiver's emotional connection to their loved one while mitigating feelings of isolation and grief. By capturing the co‐constructed meanings and intimate history between partners, AI may serve as both a coping tool during the caregiving journey and a means of maintaining connection as the spouse with dementia gradually loses cognitive function.

Ethical considerations—including consent, data privacy, and the psychological effects of AI‐mediated memory preservation—must be carefully examined to ensure that such technology complements rather than complicates the caregiving process. This proposal presents an innovative perspective on AI's role in Alzheimer's caregiving, extending beyond clinical and logistical support to address the profound emotional and relational losses experienced by spouse caregivers, ultimately offering a bridge between the past and the present in the face of memory erosion.

